# Zinc-mediated conformational preselection mechanism in the allosteric control of DNA binding to the zinc transcriptional regulator (ZitR)

**DOI:** 10.1038/s41598-020-70381-8

**Published:** 2020-08-06

**Authors:** Xinheng He, Duan Ni, Hao Zhang, Xinyi Li, Jian Zhang, Qiang Fu, Yaqin Liu, Shaoyong Lu

**Affiliations:** 1 Research Center for Marine Drugs, State Key Laboratory of Oncogenes and Related Genes, Department of Pharmacy, Renji Hospital, Shanghai Jiao Tong University, School of Medicine, Shanghai, 200127 China; 2 Department of Pathophysiology, Key Laboratory of Cell Differentiation and Apoptosis of Chinese Ministry of Education, Shanghai Jiao Tong University, School of Medicine, Shanghai, 200025 China; 3 Medicinal Chemistry and Bioinformatics Center, Shanghai Jiao Tong University, School of Medicine, Shanghai, 200025 China; 4 Department of Orthopedics, Shanghai General Hospital, Shanghai Jiao Tong University, School of Medicine, Shanghai, 200080 China

**Keywords:** Protein function predictions, Computational biology and bioinformatics, Protein structure predictions

## Abstract

The zinc transcriptional regulator (ZitR) functions as a metalloregulator that fine tunes transcriptional regulation through zinc-dependent DNA binding. However, the molecular mechanism of zinc-driven allosteric control of the DNA binding to ZitR remains elusive. Here, we performed enhanced sampling accelerated molecular dynamics simulations to figure out the mechanism, revealing the role of protein dynamics in the zinc-induced allosteric control of DNA binding to ZitR. The results suggest that zinc-free ZitR samples distinct conformational states, only a handful of which are compatible with DNA binding. Remarkably, zinc binding reduces the conformational plasticity of the DNA-binding domain of ZitR, promoting the population shift in the ZitR conformational ensemble towards the DNA binding-competent conformation. Further co-binding of DNA to the zinc–ZitR complex stabilizes this competent conformation. These findings suggest that ZitR–DNA interactions are allosterically regulated in a zinc-mediated conformational preselection manner, highlighting the importance of conformational dynamics in the regulation of transcription factor family.

## Introduction

The multiple antibiotic resistance regulator (MarR) family in bacteria contributes to virulence and survival in environmental stress^1–3^. Zinc transcriptional regulator (ZitR) and its homolog adhesin competence regulator (AdcR) are unique metalloregulatory proteins in the MarR family because most of the other members interact with DNA to restrain transcription without allosteric ligands while such function of ZitR and AdcR requires zinc binding at their allosteric sites^4,5^. Possessing two pseudotetrahedral zinc binding sites, ZitR and AdcR regulate the intake of zinc precisely in *Lactococcus lactis* and *Streptococcus pneumoniae*, respectively, contributing to their stable internal environment^3,5^.

**Figure 1:**
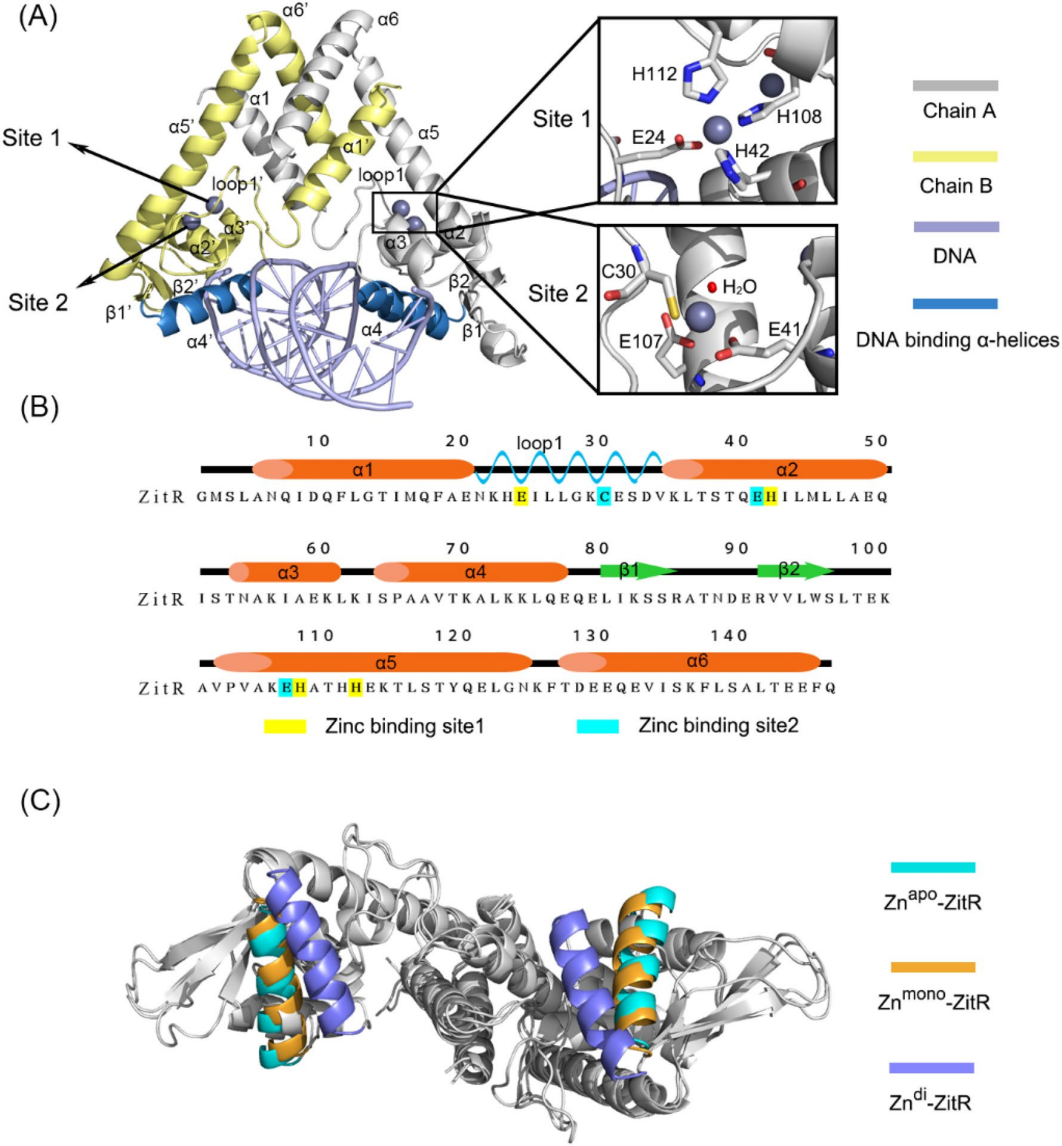
ZitR structural elements. (**A**) Cartoon representation of ZitR structure. Gray, yellow, and light blue cartoons show chain A, chain B, and DNA, respectively. Zinc is depicted by sphere models. Zoom-in image shows the coordination modes of zinc in allosteric sites. (**B**) The secondary structure information (same for chain A and B) of ZitR. (**C**) Comparison of the crystal structures of different ZitRs. Superimposed to the Zn^apo^–ZitR, structures except for the α4 and α4′ helices are colored gray. The α4 and α4′ helices are colored cyan, orange, and light purple for Zn^apo^–ZitR (PDB ID: 5YI1), Zn^mono^–ZitR (PDB ID: 5YHY) and Zn^di^–ZitR (PDB ID: 5YHX), respectively.

ZitR is a homodimer composed of two monomers in similar conformations. With 147 amino acids, each monomer comprises six α-helices, two β-sheets (also referred to as β-wings), and one important loop (named loop 1) between α1 and α2. Based on the structures of homologous proteins, the function of each secondary structural elements of ZitR is defined^6,7^. As shown in Fig. 1A, the α1 and α6 helices are involved in dimerization. The α2-α4 helices and β-wings form a winged helix-turn-helix (HTH) DNA-binding domain in which the α4 helices are responsible for DNA binding and recognition. The loop 1 and the α5 helix connect the two functional domains. In terms of allosteric sites, site 1 is composed of E24, H42, H108, and H112, while site 2 is composed of C30, E41, E107, and one water molecule (Fig. 1B)^7^.

ZitR exists in three forms in solution. During zinc deprivation, no zinc binds to ZitR (henceforth Zn^apo^–ZitR), which possess a highly dynamic and unstable feature. As shown in Fig. 1C, the distance between the two DNA binding helices reaches 44.2 Å in the crystal structure of Zn^apo^–ZitR. Accordingly, Zn^apo^–ZitR has the least affinity to DNA and permits transcription, thus such conformation is called DNA binding-incompetent conformation^8,9^. At a subpicomolar zinc concentration, ZitR dimer binds to one zinc at site 1 of each monomer (henceforth Zn^mono^–ZitR). The distance between the two DNA binding helices is 42.7 Å in the crystal structure, relatively easy to interact with DNA. Zn^mono^–ZitR interacts with the promoter DNA loosely, partially inhibiting transcription (K_DNA_ = 2.0 ± 0.2 × 10^−8^ M). Under zinc stress conditions, each monomer of ZitR binds with two zincs at sites 1 and 2, respectively (henceforth Zn^di^–ZitR). Of note, Zn^mono^–ZitR utilizes H42’s Nε2 for zinc binding but H42’s coordination atom is switched to Nδ1 in the Zn^di^–ZitR. The two zincs further decrease the distance between the two DNA binding helices, α4 and α4′, to 32.5 Å in the crystal structure, which is competent for DNA binding; thus, this conformation is called DNA binding-competent conformation. Zn^di^–ZitR has a K_DNA_ of 2.6 ± 0.4 × 10^−9^ M, leading to a strong repression of the downstream genes^5,7^. Recent determination of crystal structures of Zn^apo^–ZitR, Zn^mono^–ZitR, and Zn^di^–ZitR, as well as both Zn^mono^–ZitR and Zn^di^–ZitR in complex with DNA, has proposed that ZitR–DNA interactions are allosterically regulated by zinc in an induced-fit and prelock way^7^. Based on static crystallographic snapshots alone, however, the role of protein dynamics in this allosteric regulation may be neglected. Notably, the molecular mechanism of allostery is based on the structural dynamics of protein^10,11^. During the allosteric process, the conformational ensemble of protein is perturbated by allosteric modulators and shifts to specific conformer^12,13^. Accordingly, dynamics-based approaches are developed to explore the properties of allostery. Major methods include nuclear magnetic resonance (NMR) for atomic fluctuation^14,15^, a structure-based statistical mechanical model of allostery (SBSMMA) for allosteric communication^16–18^, and molecular dynamics (MD) simulations for conformational ensemble^19,20^. Therefore, dynamic studies of ZitR are significant and necessary to fully understand the mechanism of zinc-mediated allosteric regulation in ZitR–DNA interactions.

Accelerated molecular dynamics (aMD) simulations, which add a boost potential to the system’s potential and sample enhanced conformational space^21,22^, have been increasingly exploited to investigate long-time dynamics and infrequent conformational changes of biomacromolecules^23–27^. Simulations have also confirmed the effect of the ligand in MarR family^28^. aMD simulations of ZitR in different states can sample large-scale dynamics of ZitR, providing more complete conformational ensemble to unravel the ZitR–DNA interaction mechanism at an atomic level^29–32^. The results reveal that among the large population of Zn^apo^–ZitR conformations, only a few are deemed as DNA binding-competent. The binding of zinc elicits conformational changes through the HTH domain, resulting the stabilization of the DNA-binding domain. These effects gradually promote the population of ZitR shift towards the DNA binding-competent conformation. Notably, Zn^di^–ZitR adopts a marked conformation overlapping with that in the DNA-binding complex, contributing to its enhanced DNA binding affinity. Based on our results and previous experiments, we propose a zinc-mediated allosteric control of DNA binding to ZitR in a conformational preselection manner.

## Methods

### Construction of accelerated MD simulation system

The crystal structures of ZitR were obtained from the Research Collaboratory for Structural Bioinformatics (RCSB) Protein Data Bank (PDB)^7,33^. System composition and PDB ID of each system is shown in Table 1. To focus on the properties of wild-type ZitR, the mutated amino acids in the crystal structures were changed back to the wild-type residues.

**Table 1:** Summary of MD simulation systems.

System name	Sites occupied by zinc	DNA binding	Water molecules	Total atoms	PDB ID	PME grid sizes (Å)
Zn^apo^–ZitR	None	No	26,812	85,260	5YI1	108 × 108 × 108
Zn^apo^–ZitR–DNA	None	Yes	16,482	71,854	5YI3^a^	96 × 96 × 96
Zn^mono^–ZitR	Site 1	No	26,435	84,127	5YHY	108 × 108 × 108
Zn^mono^–ZitR–DNA	Site 1	Yes	16,482	55,240	5YI3	96 × 96 × 96
Zn^di^–ZitR	Sites 1&2	No	21,951	70,685	5YHX	100 × 100 × 100
Zn^di^–ZitR–DNA	Sites 1&2	Yes	15,664	52,804	5YI2	90 × 90 × 90

^a^Zn^apo^–ZitR–DNA system was constructed by the deletion of zinc ions of Zn^mono^–ZitR–DNA system.

### System preparations

MD simulations of ZitR were performed with the AMBER 14 program^.^ The amber ff14SB force field^34^ were assigned for the ZitR protein and DNA. Zinc ions were treated by a four-point charge model as previously reported^35,36^. The complexes were first solvated in an orthorhombic TIP3P water box^37^. Then, after Cl^-^ was added to the system for neutralization, NaCl equivalent to the physiological saline concentration (0.15 mol/L) were added. The Particle Mesh Ewald (PME) method was conducted in all systems to treat long-range interactions, while a cutoff was set at 10 Å in order to deal with electrostatics in short range and van der Waals forces^38^. Sizes of the PME grid were chosen based on the system volume and the grid spacing was 1 Å. The SHAKE algorithm was employed to constrain hydrogen-containing bonds^39^. Information of the six systems is also shown in Table 1.

### Conventional MD (cMD) simulations

After preparations, all systems underwent two rounds of energy minimization. In the first process, the protein, DNA and zincs were confined with a positional restraint of 500 kcal mol^−1^ Å^−2^. Other molecules were minimized in 2,000 steps of steepest descent minimization cycles, then 3,000 steps of conjugate gradient minimization cycles. In the second process, the whole systems were minimized without any restriction; the steepest descent method was first employed for 4,000 cycles, next, the conjugated gradient method was applied for the subsequent 6,000 cycles. After minimization, the systems were heated gradually from 0 to 300 K in 300 ps, during which the ZitR complex were constrained with a positional restraint of 10 kcal mol^−1^ Å^−2^ in a canonical ensemble (NVT). Next, NVT equilibration runs of the systems were carried out at 300 K for 700 ps, with a positional restraint of 10 kcal mol^−1^ Å^−2^ in ZitR, DNA and zincs. Then, 50 ns MD simulations with a timestep of 2.0 fs were employed at 1 atm pressure and approximately 300 K with the AMBER 14 package to get necessary energy data for aMD. System temperature was controlled using Langevin dynamics with a collision frequency of 1 ps^−1^.

### Accelerated MD simulations

After cMD simulations, the systems underwent accelerated molecular dynamic (aMD) simulations to be sampled more completely. During aMD, E_thresh_ is defined as the lower boundary of the potential energy surface, while an energy value above E_thresh_ is the “boost energy”. As shown in Eqs. (1) and (2), if the potential energy in the present step, V(r), was lower than E_thresh_, it would be increased by a value of ΔV(r). On the other hand, if instantaneous potential energy was greater than or equal to E_thresh_, it would retain its value. The applied potential energy in the aMD simulation was defined as V^*^(r).

1 $${\text{V}}^{*} \left( {\text{r}} \right) \, = {\text{ V}}\left( {\text{r}} \right), {\text{V}}\left( {\text{r}} \right) \, \ge {\text{E}}_{{{\text{thresh}}}}$$

2 $${\text{V}}^{*} \left( {\text{r}} \right) \, = {\text{ V}}\left( {\text{r}} \right) \, + \Delta {\text{V}}\left( {\text{r}} \right), {\text{V}}\left( {\text{r}} \right) \, < {\text{E}}_{{{\text{thresh}}}}$$

The bias potential decreases energy barriers and flattens the potential energy surface, resulting in the acceleration of conversion between the low-energy conformers^40,41^. With the ΔV(r) defined in Eq. (3), indispensable details of the potential energy surface were kept.

3 $$\Delta {\text{V}}\left( {\text{r}} \right) \, = \frac{{\left( {E_{thresh} - V\left( r \right)} \right)^{2} }}{{E_{thresh} - V\left( r \right) + \alpha }}$$

As shown in Eq. (3), the extent of aMD was depended on the energy threshold E_thresh_ and the acceleration parameter $\alpha$. Both increasing E_thresh_ and decreasing $\alpha$ result in an enhanced sampling^41^. For our systems, a “dual boost’’ protocol was employed. The parameters of two potentials were determined on Eqs. (4)–(7). Among them, E_threshP_ represents the energy threshold for potential energy, while E_threshD_ means energy threshold for dihedral energy. The α_P_ means the acceleration parameter α for potential energy and α_D_ is for dihedral energy. E_tot avg_ and E_dih avg_ are the average total potential energy and average dihedral energy obtained from the cMD trajectories, respectively. N_atoms_ and N_residues_ stand for the number of atoms and residues in the systems.

4 $${\text{E}}_{{{\text{threshP}}}} = {\text{ E}}_{{\text{tot avg}}} + {\text{ N}}_{{{\text{atoms}}}} \times \, 0.{16}\,{\text{kcal}}/{\text{mol}}$$

5 $$\alpha_{{\text{P}}} = {\text{ N}}_{{{\text{atoms}}}} \times \, 0.{16}\,{\text{kcal}}/{\text{mol}}$$

6 $${\text{E}}_{{{\text{threshD}}}} = {\text{ E}}_{{\text{dih avg}}} + {\text{ N}}_{{{\text{residues}}}} \times { 4}\,{\text{kcal}}/{\text{mol}}$$

7 $$\alpha_{{\text{D}}} = {\text{ N}}_{{{\text{residues}}}} \times { 4}/{5}\,{\text{kcal}}/{\text{mol}}$$

From the last structures of cMD simulations, 500 ns aMD simulations were carried out for the systems. Van der Waals forces and electrostatic interactions were computed in the same way as cMD processes. SHAKE algorithm was also applied to all bonds including hydrogen^39^. The environments of systems were kept the same as cMD simulations.

### Calculation of potential of mean force (PMF)

PMF was calculated to observe the conformational ensemble in ZitRs. For every snapshot in each aMD simulation trajectory, root-mean-square deviation (RMSD) value of the DNA binding domain (from the α2 to the β2) was calculated referring to Cα atoms of the starting structure of ZitR without DNA and the distance between the α4 and α4′ helices was defined as the distance of Cα atoms between A71 and A71′. Then, the RMSD and distance values were set as two reaction coordination (x, y). Using the following Eq. (8), the PMF values were calculated.

8 $$\Delta {\text{G}}\left( {{\text{x}},{\text{ y}}} \right)\, = \,{\text{k}}_{{\text{B}}} T{\ln}g\left( {{\text{x}},{\text{ y}}} \right)$$

In (8), k_B_ means the Boltzmann constant, *T* is the temperature of systems (300 K) and *g*(x, y) represents the normalized joint probability distribution. Meanwhile, the minimum energy value was set to zero. 30 bins were applied to generate the landscape in both x and y directions.

To obtain representative structures shown in Fig. 4, we firstly defined areas that have lower free energy than any area around them as the energy basins. Then, we obtained the coordinates of energy basins and found their corresponding snapshots in trajectories. The representative structures were obtained by the cluster module provided by Amber Suite.

### Dynamical network analysis

The dynamic cross correlation matrix (DCCM) was applied to analyze the interaction between ZitR residues. The correlation coefficient C_ij_ was calculated according to Eq. (9).

9 $${\text{C}}_{{{\text{ij}}}} \, = \,\left( {\Delta {\text{r}}_{{\text{i}}} \, \times \,\Delta {\text{r}}_{{\text{j}}} } \right)/\left( {\left\langle {\Delta {\text{r}}_{{\text{i}}}^{{2}} } \right\rangle \, \times \,\left\langle {\Delta {\text{r}}_{{\text{j}}}^{{2}} } \right\rangle } \right)^{{{1}/{2}}}$$

In the equation, Δr_i_ and Δr_j_ mean the atomic displacement vectors for Cα atoms i and j, respectively. The angle brackets represent the average calculation among the time of simulations. Collected in a colored matrix form, C_ij_ can be regarded as a measurement of the fluctuation correlation between residues.

The ZitR community networks were defined as sets of nodes connected by C_ij_ weighted edges. The Cα atom of each residue was considered as one node. Two nodes would be connected by edges if the distance between the corresponding residues were within a 4.5 Å cutoff for at least 75% of the aMD trajectory. Using the Floyd–Warshall algorithm^42^, the optimal paths between all pairs of nodes were calculated. Then, the number of pairwise optimal paths was regarded as the betweenness of an edge. A community was composed of nodes that are more densely interconnected with each other than to nodes in other communities. With the help of the Girvan–Newman algorithm, the distribution of communities was optimized to maximize the modularity measure^43^. Communities whose residues are less than three were discarded. Connectivity between communities was measured by the betweenness value.

## Results

### The extensive conformational distribution of Zn^apo^–ZitR

**Figure 2:**
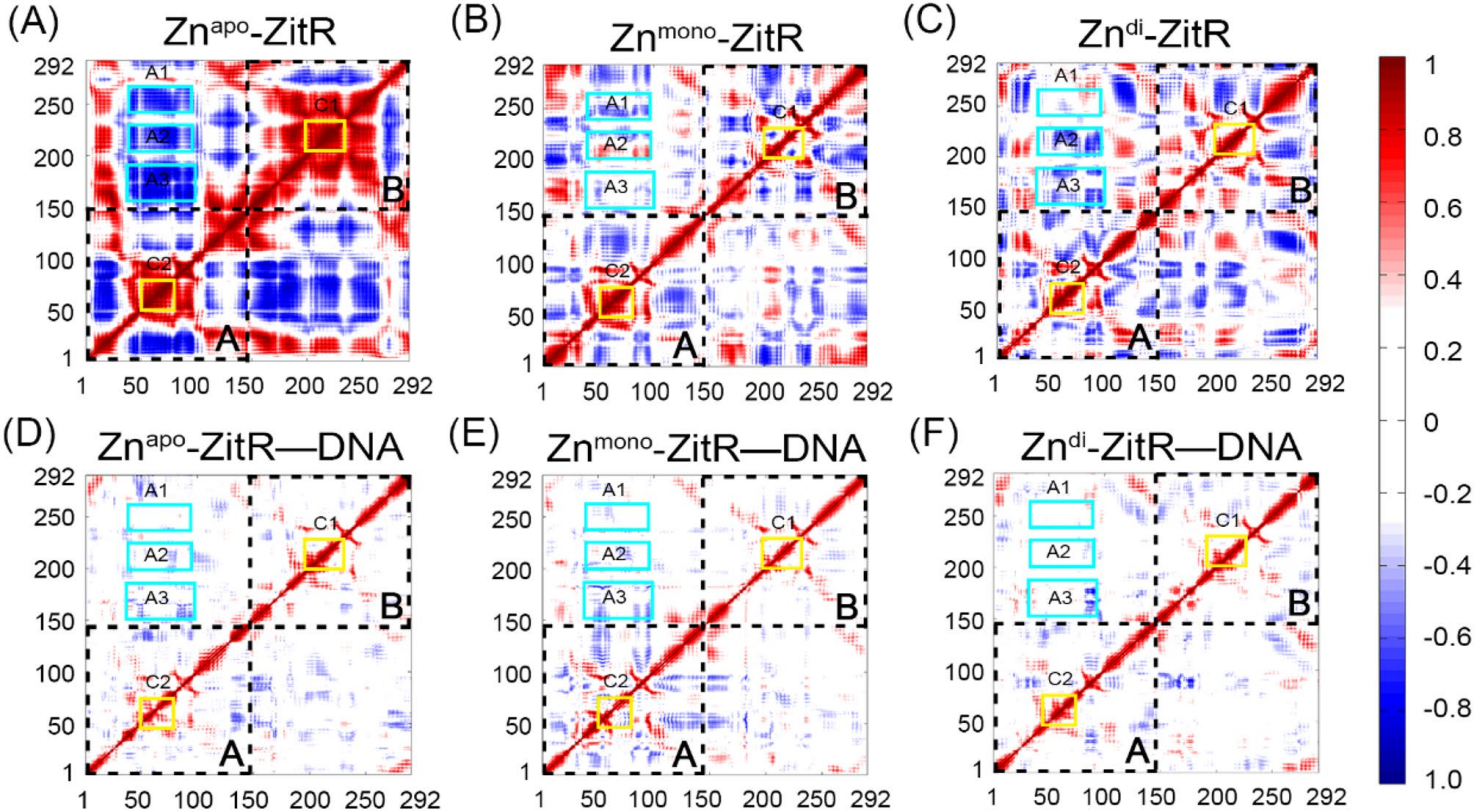
The dynamic cross-correlation matrixes (DCCM) of the six systems: Zn^apo^–ZitR (**A**), Zn^mono^–ZitR (**B**), Zn^di^–ZitR (**C**), Zn^apo^–ZitR–DNA (**D**), Zn^mono^–ZitR–DNA (**E**), and Zn^di^–ZitR–DNA (**F**). Areas of chains A and B are separated by dashed lines. Red and blue express correlations and anticorrelations, respectively. Yellow rectangles show areas with obvious correlations, while cyan rectangles show areas with obvious anticorrelations. The interactions possessing an absolute correlation coefficient of less than 0.3 are neglected for clarity.

Recent experimental studies of Zn^apo^–ZitR have suggested that it always adopts a DNA binding-incompetent conformation^7^. To probe the conformational ensemble of Zn^apo^–ZitR in solution, DCCM and free energy landscape analyses of ZitR were performed. Overall, as shown in Fig. 2A, both intra- and inter-chain motions between residues are the most correlation in the Zn^apo^–ZitR. Namely, zinc- or DNA-binding of ZitR restrains interactions between residues (Fig. 2B–F). Remarkably, the C1 and C2 areas in Fig. 2A represent the intra-chain motions, reflecting correlations between the α3, α4, and β1. The A1–A3 areas represent the inter-chain motions, showing anticorrelations between the winged HTH DNA-binding domain of chains A and B, especially for the α4 and α4′ helices. In fact, the strong correlation or anti-correlation motions between residues in the Zn^apo^–ZitR are localized in the region of the DNA binding domain. These observations indicate that the DNA binding domain of Zn^apo^–ZitR has significant conformational flexibility owing to the absence of zinc or DNA binding. Experimentally, metal-unbound states of metalloregulators exhibited increased flexibility compared with metal-bound states^44^, further supporting our computational results.

**Figure 3:**
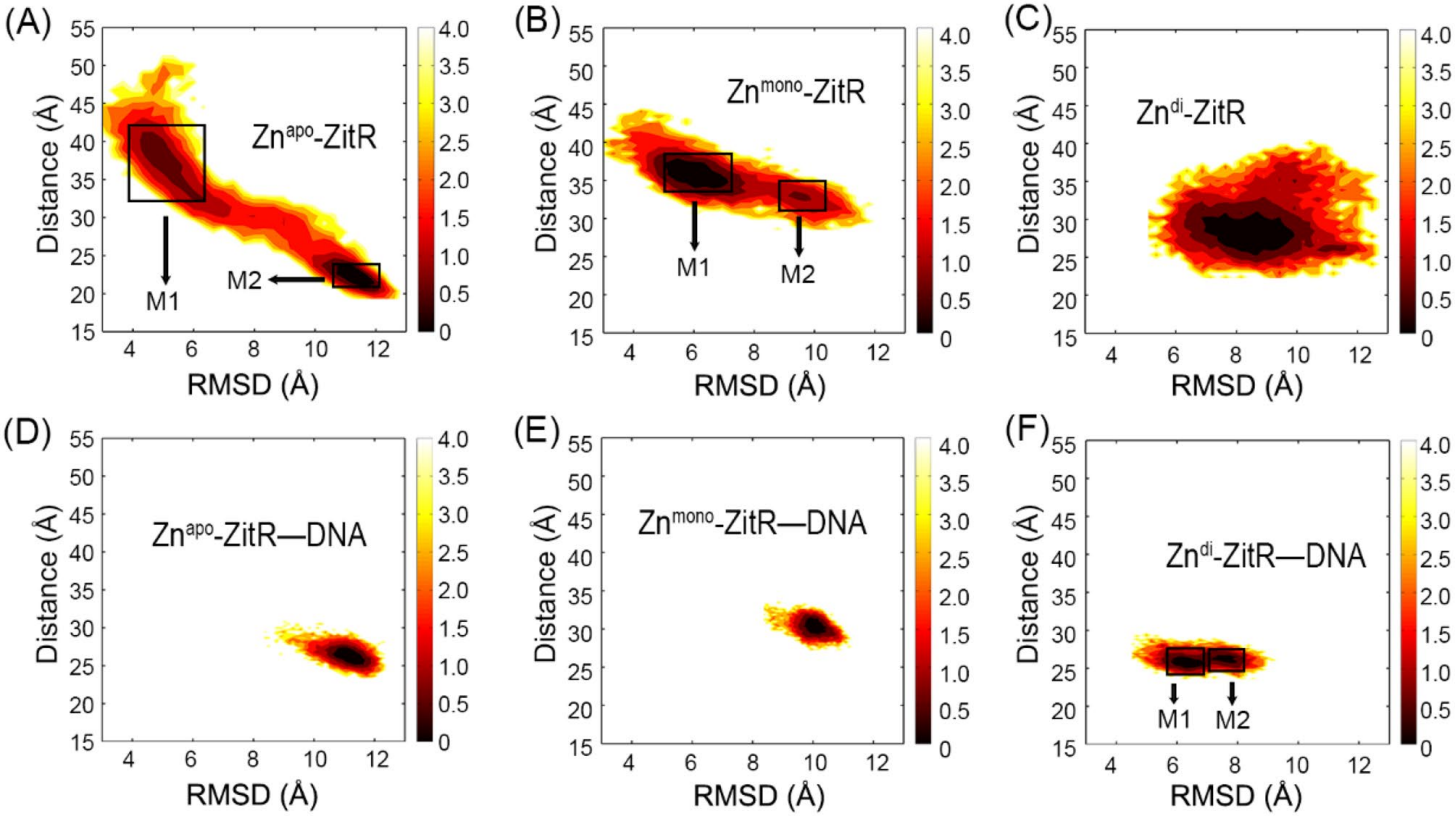
The free energy landscapes of Zn^apo^–ZitR (**A**), Zn^mono^–ZitR (**B**), Zn^di^–ZitR (**C**), Zn^apo^–ZitR–DNA (**D**), Zn^mono^–ZitR–DNA (**E**), and Zn^di^–ZitR–DNA (**F**) complexes. The unit of PMF is kcal/mol. Rectangles represent the major conformers (M1 and M2) of Zn^apo^–ZitR, Zn^mono^–ZitR, and Zn^di^–ZitR–DNA in A, B, and F, respectively. M1 and M2 are defined as an area that has a color deeper than the surrounding color and is not connected to each other.

**Figure 4:**
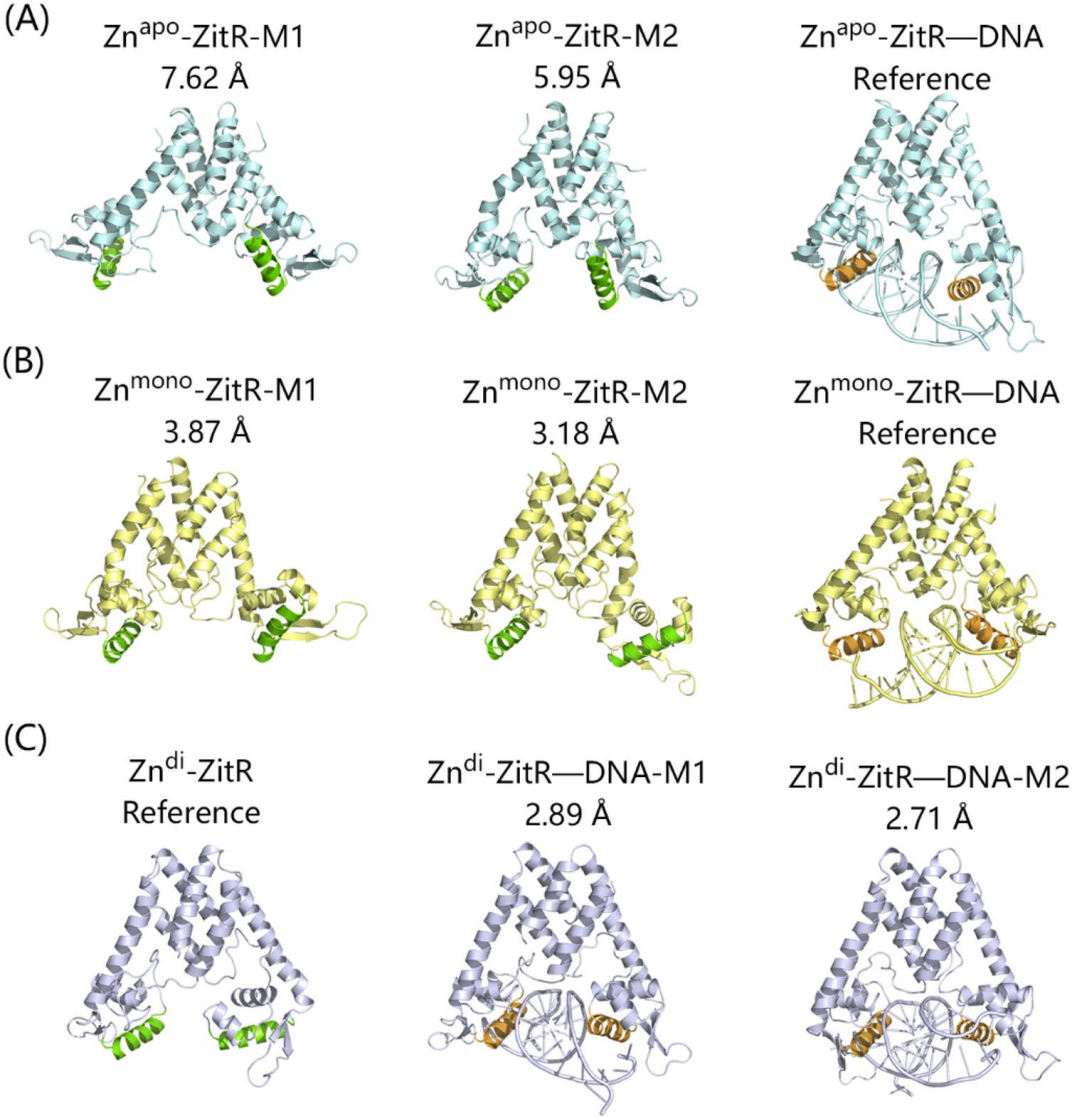
Structures of major conformations of Zn^apo^–ZitR (**A**), Zn^mono^–ZitR (**B**), and Zn^di^–ZitR (**C**). Chain A and B are located on the left and right sides, respectively. α4 and α4′ helices are colored as green cartoons without DNA binding and as orange cartoons when binding to DNA. Numbers represent the RMSD values when superimposed to the reference structures.

The free energy landscapes were also analyzed to monitor ZitR conformational ensembles using the following two parameters. One is the RMSD of DNA binding domain referred to the starting structure of ZitR without DNA to compare the difference between ZitR and ZitR–DNA directly, and the other is the distance between the α4 and α4′ helices. As shown in Fig. 3A, Zn^apo^–ZitR has an extensive conformational distribution and two major conformations, M1 and M2, are observed in the Zn^apo^–ZitR. The representative structures for the M1 and M2 were clustered to explore their structural properties. As shown in Fig. 4A, M1 of Zn^apo^–ZitR is relatively loose, while M2 is compact and close to the DNA binding structure. However, the directions of α4 and α4′ helices in both M1 and M2 are distinct from those of Zn^apo^–ZitR–DNA, reflected by large RMSD values. Hence, both M1 and M2 of Zn^apo^–ZitR represent DNA binding-incompetent conformations. It can be inferred that successful DNA binding requires substantial changes in the major conformers of Zn^apo^–ZitR. Moreover, the RMSD of Zn^apo^–ZitR varies from 3 to 12 Å in Fig. 3A, suggesting large fluctuations of its DNA binding domain. The distance between the α4 and α4′ helices varies from 20 to 50 Å in the Zn^apo^–ZitR. Thus, Zn^apo^–ZitR has a wide conformational distribution. However, the distance for the DNA binding-competent conformation is approximately 30 Å, occupying only a small part of Zn^apo^–ZitR conformations^7^. Of note, Zn^apo^–ZitR has a small conformational overlap with Zn^apo^–ZitR–DNA but no overlap in major conformations (Figs. 3A,D, 4A), suggesting that the conformational ensemble of Zn^apo^–ZitR contains rare DNA binding-competent conformation.

### Zinc^mono^–ZitR has a population shift tendency

Given that the conformational ensemble of Zn^apo^–ZitR contains a few DNA binding-competent conformations, we next investigated the allosteric effect of zinc on the conformational ensemble of ZitR. Analyses of the C1 and C2 correlations show that the motions of the DNA binding domain in the Zn^mono^–ZitR decrease with one zinc (Fig. 2A,B). Remarkably, the A1–A3 anticorrelations are reduced in the Zn^mono^–ZitR compared to those in the Zn^apo^–ZitR. These results suggest that zinc binding greatly impairs correlation motions of ZitR.

Free energy landscape analyses reveal that compared to the wide conformational distribution in the Zn^apo^–ZitR (Fig. 3A), the binding of zinc narrows down the conformational distribution in the Zn^mono^–ZitR (Fig. 3B). Same as the Zn^apo^–ZitR, the Zn^mono^–ZitR also exists two major conformers. However, the distribution of the two conformers is confined in the presence of zinc. Further binding of DNA to the Zn^mono^–ZitR restrains the correlation motions of the DNA binding domain (Fig. 2E) and generates only one major conformation (Fig. 3E). A comparison of conformational distribution in the Zn^mono^–ZitR and Zn^mono^–ZitR–DNA shows that the two systems have a partial conformational overlap involving the M2 of Zn^mono^–ZitR and the major conformation of Zn^mono^–ZitR–DNA. From the structural standpoint (Fig. 4B), M1 and M2 conformers of Zn^mono^–ZitR are relatively similar to Zn^mono^–ZitR–DNA, with an RMSD of 3–4 Å. From M1 to M2, the most important change is located at the DNA binding domain of chain B, which resembles the DNA binding-competent conformation. Because M2 conformer is relatively close to Zn^mono^–ZitR–DNA, it suits DNA binding more but can still be improved in the similarity between it and DNA binding-competent conformation. Taken together, zinc binding shifts the population towards the DNA binding-competent conformation and DNA binding to Zn^mono^–ZitR further stabilizes this state.

### Zn^di^–ZitR has a high proportion of DNA binding-competent conformation

Considering that one zinc binding to ZitR can partially shift the conformational ensemble of ZitR, we then investigated the effect of two zincs binding on the population of ZitR. Comparison of the C1 and C2 correlations and the A1–A3 anticorrelations in Fig. 2A–C indicates that intra- and inter-chain motions are more restrained in response to two zinc ions. Compared to Zn^mono^–ZitR, the C2 correlation and the A1 anticorrelation of Zn^di^–ZitR are decreased due to the second zinc. Thus, binding of the second zinc further leads to the decline of motions between residues and renders the conformation of ZitR similar to the stable DNA binding-competent state (Fig. 2D–F).

In terms of the PMF free energy landscapes, Zn^di^–ZitR has a markedly confined conformational distribution compared to Zn^apo^–ZitR and Zn^mono^–ZitR. It exists only one major conformation in solution. DNA binding further restrains the conformational dynamics of Zn^di^–ZitR, exemplified by the decrease of motions between residues (Fig. 2F) and the confined conformational ensemble (Fig. 3F). The conformational ensemble of Zn^di^–ZitR**–**DNA largely overlaps with Zn^di^–ZitR, even in the major conformations (Fig. 3C,F), confirming the DNA binding ability of Zn^di^–ZitR. As shown in Fig. 4C, the RMSD between Zn^di^–ZitR and the M1 of Zn^di^–ZitR**–**DNA is 2.89 Å, while the RMSD between Zn^di^–ZitR and the M2 of Zn^di^–ZitR**–**DNA is 2.71 Å. Therefore, Zn^di^–ZitR has the smallest RMSD to the Zn^di^–ZitR**–**DNA compared to the Zn^apo^–ZitR and Zn^mono^–ZitR, indicative of the similarity among major conformers of Zn^di^–ZitR and Zn^di^–ZitR**–**DNA. Regardless of the overall structure or the α4 and α4′ helices, Zn^di^–ZitR is obviously close to the DNA binding-competent state. In conclusion, the second zinc binding further shifts the population of ZitR to the DNA binding-competent conformation and DNA binding stabilizes this conformation. Similar population shift effects have also been observed in other MarR proteins^28^.

### Zinc binding promotes the intermolecular interactions of ZitR–DNA complex

**Figure 5:**
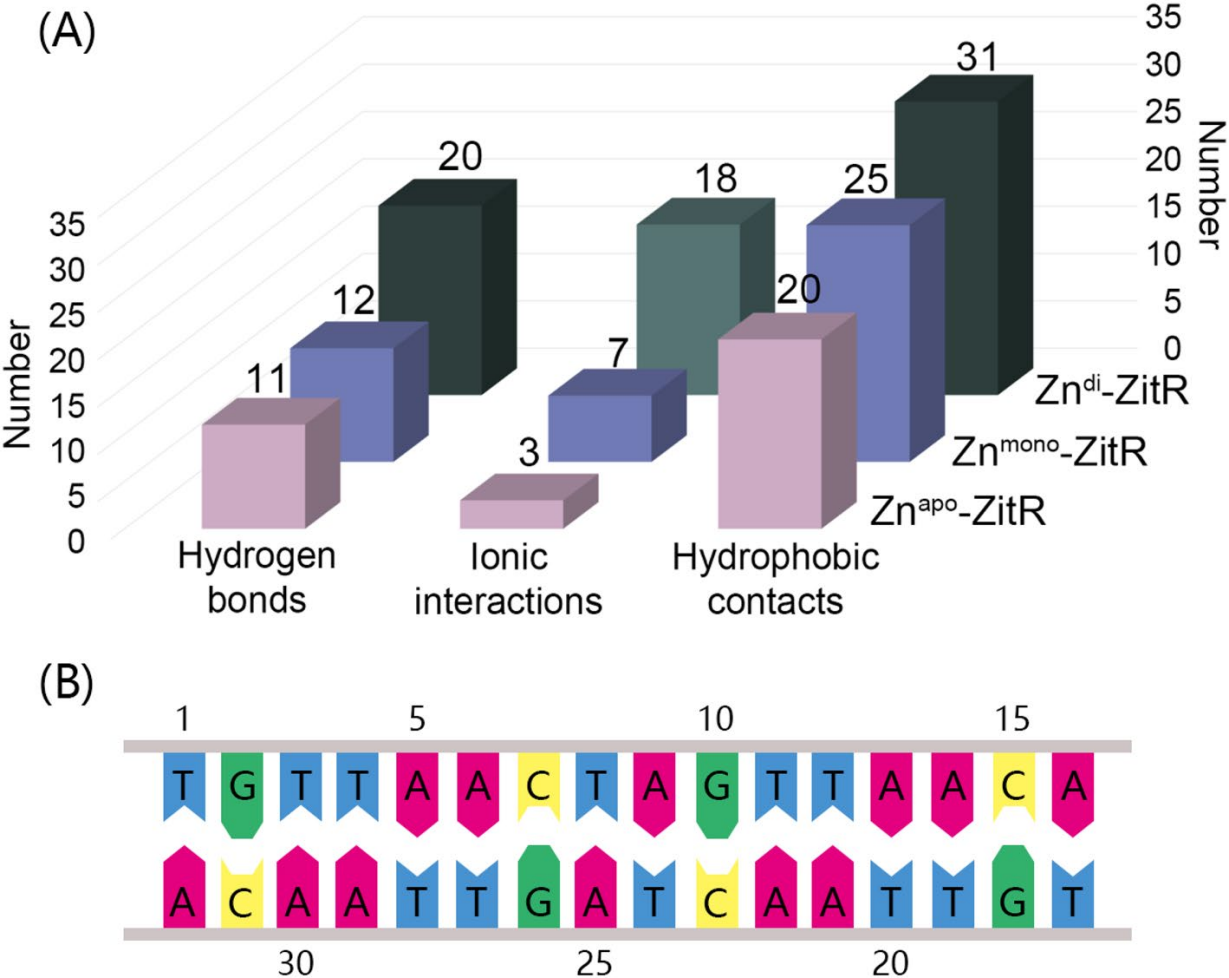
ZitR–DNA interactions. (**A**) Histograms of intermolecular interactions between DNA and different ZitR states. (**B**) The DNA sequence of our ZitR–DNA systems.

The intermolecular interactions such as hydrogen bonds, ionic interactions, and hydrophobic contacts serve an important role in the DNA–protein interaction^45,46^. Therefore, based on the representative structures of ZitR**–**DNA complexes in Fig. 4, we calculated the intermolecular interactions between DNA and ZitRs. As shown in Fig. 5, with the increase of zinc binding, there are more intermolecular interactions between ZitR and DNA, especially in ionic interactions. Compared to both Zn^apo^–ZitR and Zn^mono^–ZitR, Zn^di^–ZitR has much more hydrogen bonds between DNA and ZitR. Most of them are formed between oxygen atoms of phosphate-deoxyribose backbone and hydrogen bond donors of ZitR, such as G10 and H23 in Zn^apo^–ZitR (Supplementary Fig. S1), T24 and K70′ in Zn^di^–ZitR (Supplementary Fig. S2). Ionic interactions comprise electrostatic attraction between the DNA backbone and basic amino acid. For example, side chains of H42′ (Zn^mono^–ZitR, Supplementary Fig. S3) and H23′ (Zn^di^–ZitR, Supplementary Fig. S4) both interact with G26 in this way. In terms of the number of ionic interactions, Zn^apo^–ZitR only possesses 3 ionic interactions between DNA and ZitR. This number rises to 7 and 18 in Zn^mono^–ZitR and Zn^di^–ZitR, respectively. Hydrogen bonds and ionic interactions are germane in the backbone of DNA, so the DNA sequence changes cause a limited influence on them.

Hydrophobic contacts mainly generate from the tendency of hydrophobic molecules to approach each other, namely the ring of deoxyribose and nucleobase tends to reach non-polar amino acids. For instance, T1 is close to V93′ in Zn^mono^–ZitR, preventing surrounding water from reaching (Supplementary Fig. S5). Of note, thymine, including T1, T3, T11, T17, T20, T24, T27, and T28, are all involved in hydrophobic interactions. Thus, it is implied that ZitR is likely to bind to a DNA sequence with more adenine and thymine. With the addition of zincs, the number of hydrophobic contacts also increases. As a result, Zn^apo^–ZitR has a weak interaction with DNA, Zn^mono^–ZitR can moderately interact with DNA, and Zn^di^–ZitR can bind with DNA strongly.

Mutagenesis and NMR experiments of AdcR has proved the utility of L81 and L57 in the conformational entropy, while V34, L4, and I16 serve important roles in conformational exchange^47^. From the perspective of ZitR, K73 and K74 at the α4 and α4′ helices form multiple hydrogen bonds and ionic interactions with T20 (Supplementary Fig. S6) and T8 (Supplementary Fig. S7), respectively, while A66 at the α4 and α4′ helices have two hydrophobic contacts with T27 and T28 (Supplementary Fig. S8). It is confirmed that the α4 and α4′ helices are the key secondary structure for DNA binding of ZitR. In addition, these residues may occupy an important role in sequence specificity and transcription inhibition.

From a perspective of dynamic, time-dependent formations of these interactions were also analyzed in different ZitRs for a comprehensive understanding of the relationship between ZitR and DNA. As shown in Supplementary Fig. S9, hydrogen bonds in Zn^di^–ZitR outnumber that in Zn^apo^–ZitR and Zn^mono^–ZitR. Also, Zn^mono^–ZitR has more hydrogen bonds in the last 200 ns than Zn^apo^–ZitR, indicating that Zn^mono^–ZitR binds DNA more tightly after equilibrium. In terms of ionic interactions in Figure S10, Zn^apo^–ZitR has approximately five ionic interactions and fluctuates a lot, verifying instability of the complex. The number of ionic interactions in Zn^mono^–ZitR is around 10 while which of Zn^di^–ZitR can reach 20 or higher, proving that ZitR with zinc interacts with DNA well. More ionic interactions also benefit the high DNA binding affinity of Zn^di^–ZitR. At last, Zn^apo^–ZitR, Zn^mono^–ZitR, and Zn^di^–ZitR tend to have a hydrophobic contact number of less than 20, around 20, and more than 25, respectively (Supplementary Fig. S11). Hence, zinc also increases the overall hydrophobic contacts between ZitR and DNA, especially in the last 100 ns of Zn^di^–ZitR. Collectively, time-dependent interactions prove the promoting effect of zinc in ZitR–DNA complexes.

### Allosteric signal analysis of ZitR

**Figure 6:**
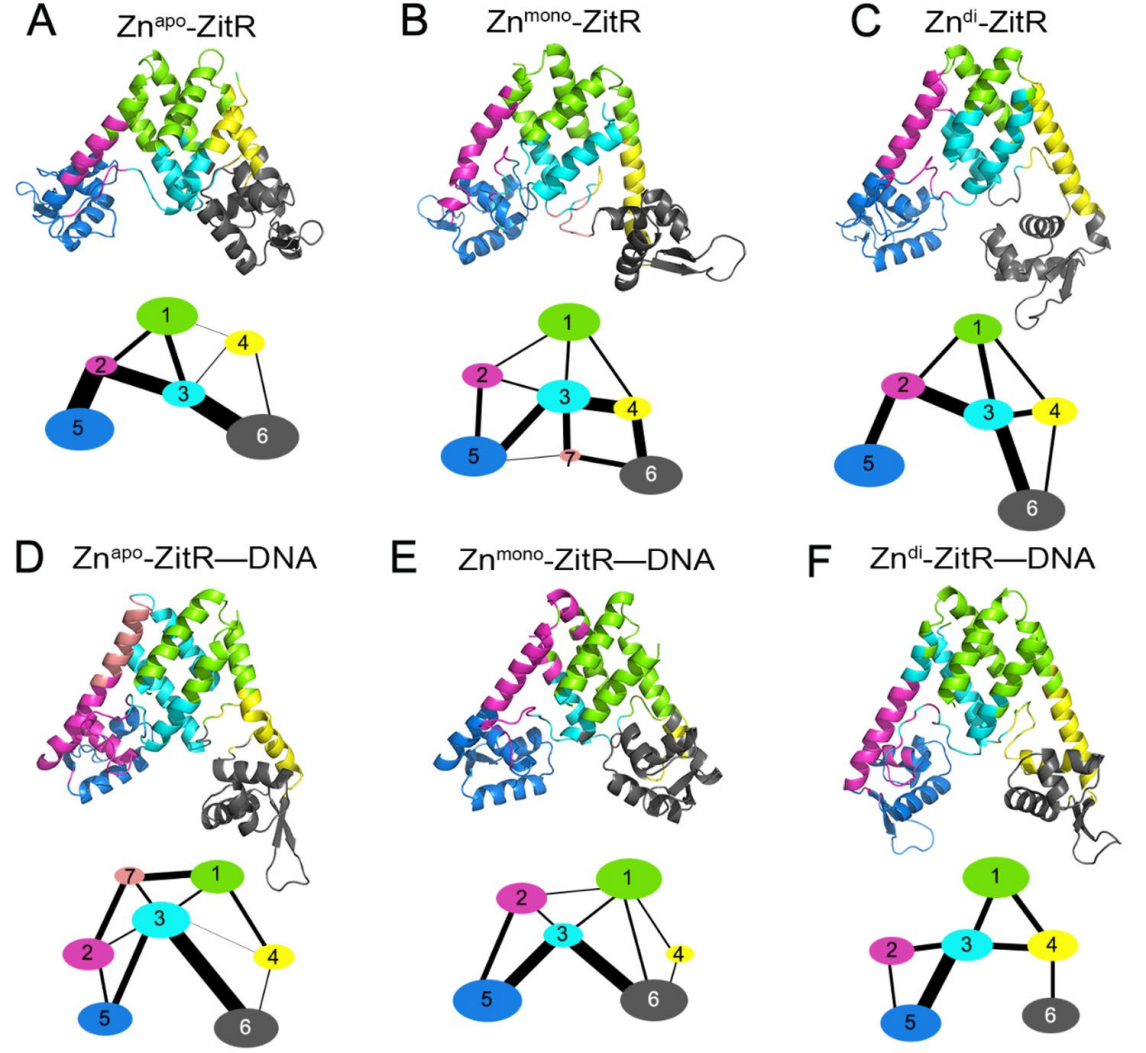
Colored community networks of Zn^apo^–ZitR (**A**), Zn^mono^–ZitR (**B**), Zn^di^–ZitR (**C**), Zn^apo^–ZitR–DNA (**D**), Zn^mono^–ZitR–DNA (**E**), and Zn^di^–ZitR–DNA (**F**). Color scheme: community 1 (C1, green), community 2 (C2, magenta), community 3 (C3, cyan), community 4 (C4, yellow), community 5 (C5, blue), community 6 (C6, black) and community 7 (C7, salmon). Each oval represents an individual community. The area of it is in direct proportion to the number of its residues. The widths of lines connecting ovals are proportional to the betweenness.

A more complete analysis of allosteric communication is necessary to elucidate the crucial pathways of allosteric signal propagation in ZitR. To reveal the zinc-induced allosteric mechanism, we firstly used community analysis which provides clues about allosteric communications. In the analysis, the community structures typically reveal communities of nearby residues, although they may be distant in sequence. The networks of ZitR were split into communities by the Girvan–Newman algorithm. To clarify the community networks, structural domains corresponding to their communities were illustrated with consistent colors. A coarse-grained representation of ZitR communities is depicted in Fig. 6. The width of the bonds connecting communities is proportional to the highest score in edge connecting communities.

In the community network (Fig. 6), C3 is a significant community located at the center and connects to most other communities. This community contains the second half of the helix α1 and helix α1′, the first half of loop1 and loop1′, as well as a part of zinc binding site 1. C5 and C6 are composed of residues from α4 and α4′ helices. Thus, interactions between C3 and C5/C6 are crucial for the propagation of the allosteric signal. Holistically, the addition of zinc generally attenuates the communications between C5 and C6 communities. From Zn^apo^–ZitR (Fig. 6A) to Zn^mono^–ZitR (Fig. 6B), the pathway through the C3 community is significantly dampened due to zinc binding but Zn^mono^–ZitR utilizes C7 community, a section of loop1, to partially compensate it. From Zn^mono^–ZitR (Fig. 6B) to Zn^di^–ZitR (Fig. 6C), the pathway through C3 is similar in strength, whereas the absence of C7 leads to increasing difficulty for ZitR to transmit signals between C5 and C6 communities. These observations suggest that zinc binding restrains the movement of helix α4 by weakening signal transmission between the α4 and α4′ helices. According to a previous NMR research, when dynamics of key structures are restricted, metalloregulatory MarR tends to take on DNA binding-competent conformation^47^. Thus, the decrease of signal transmission between the α4 and α4′ helices implies the increase of DNA binding affinity. Binding of DNA to different zinc-bound ZitR further attenuates community communications (Fig. 6D–F), in line with the results of correlation motion analyses and PMF free energy landscape analyses that `DNA binding stabilizes the conformation of ZitR.

Besides community analysis, we also applied the AlloSigMA web server on the structure of Zn^apo^–ZitR, Zn^mono^–ZitR, and Zn^di^–ZitR to quantitatively predict the allosteric communication in ZitR^18,48^. AlloSigMA is based on SBSMMA and broadly used in the field of allosteric effect evaluation^16,30,49^. Originated by the zinc sites, allosteric effects are successfully transmitted to DNA-binding helices, and corresponding allosteric free energies match the sequence of our community analysis (Δg of Zn^apo^–ZitR = 0.307 kcal/mol, Δg of Zn^mono^–ZitR = 0.079 kcal/mol, and Δg of Zn^di^–ZitR = 0.036 kcal/mol). It confirms that the addition of zinc binding allosterically quenches the dynamics of the DNA-binding domain.

## Discussion

**Figure 7:**
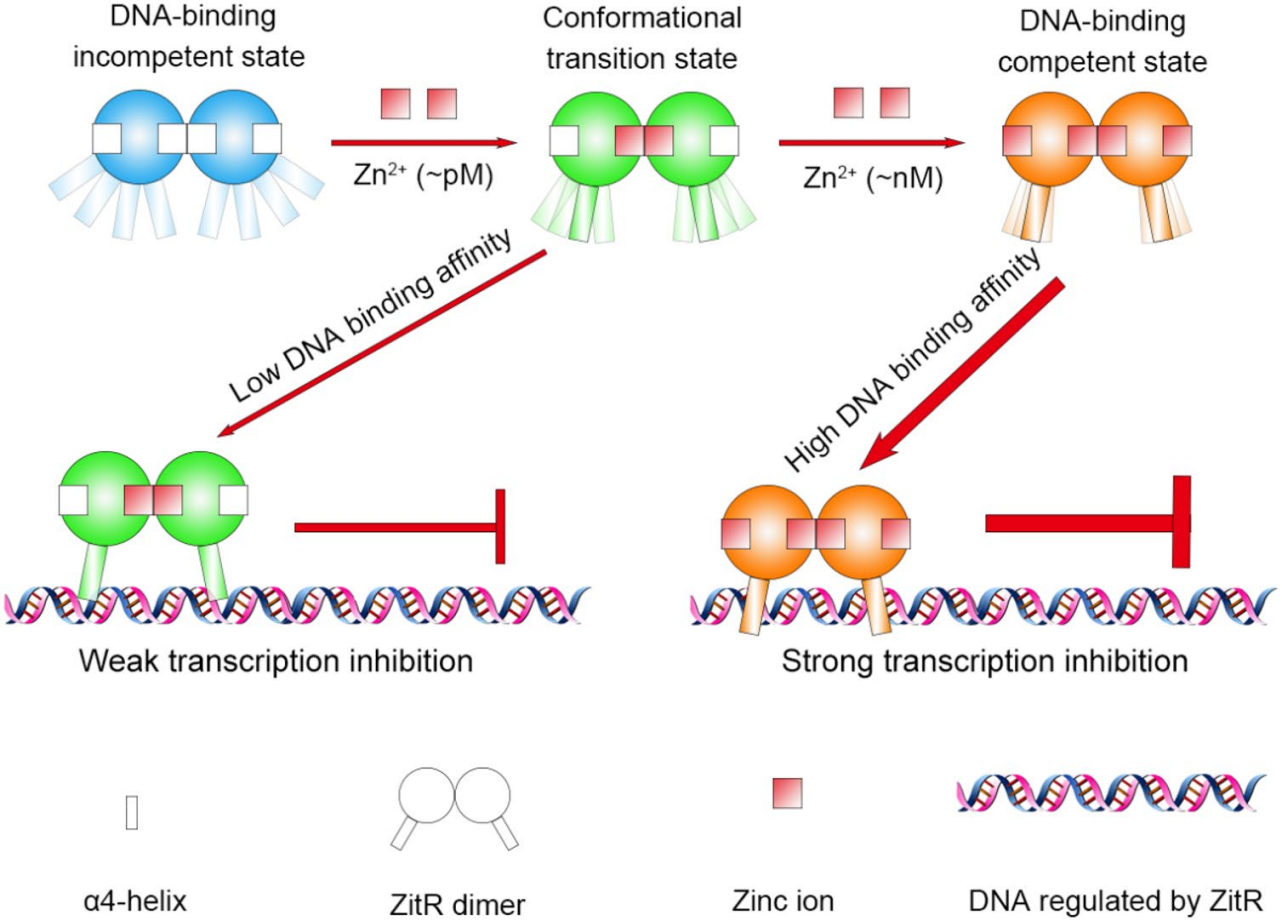
Model for zinc-mediated conformational preselection mechanism in ZitR–DNA interaction. Multiple rectangles represent possible locations taken by the α4 and α4′ helices. Lower transparency means a higher probability of occurrence.

Here, we performed aMD simulations on a MarR family protein and exploit the dynamic model to elucidate the DNA-binding mechanism of wild-type ZitR. Based on the analyses of aMD simulations and previous experiments, we proposed a conformational preselection mechanism in the ZitR–DNA interaction that is allosterically regulated by zinc. As shown in Fig. 7, under the zinc starvation condition, ZitR binds with no zinc, but it binds with one zinc and two zincs in the situation that the concentration of zinc raises to pM and nM, respectively. Zinc quenches the motion of the DNA binding domain (mainly the α4 and α4′ helices) and renders the population shift towards DNA binding-competent conformation. Due to the extensive conformational distribution, Zn^apo^–ZitR is difficult to reach DNA binding-competent conformation so it exhibits a low binding affinity for DNA. However, Zn^apo^–ZitR contains a few conformations that are possible to interact with DNA. Upon binding of one zinc, the conformational ensemble of Zn^mono^–ZitR has relatively shifted to DNA binding-competent conformation but its α4 and α4′ helices still fluctuate to a certain degree. In terms of Zn^di^–ZitR, its major conformation highly overlaps with the DNA binding-competent conformation, in concert with its highest DNA binding affinity. Moreover, ZitR tends to bind to the DNA with more adenine and thymine. These two bases are abundant (74.6%) in the zit_p_ domain of *Lactococcus lactis,* the binding site of ZitR^5^. In particular, ZitR has been shown to interact with the conserved and A-T-rich palindromic **TTAAC**YR**GTTAA** operator domain^5,50,51^. These phenomena partially explain the adenine and thymine preference in a co-evolving way. It is also observed that zinc promotes the interactions between ZitR and DNA and inhibits the motions and interactions between DNA binding communities, which is also proved by AlloSigMA. Thus, zinc generally preselects the α4 and α4′ helices at the DNA binding-competent conformation and enhances the DNA binding affinity of ZitR.

These computational discoveries extend the traditional concept of protein dynamics in MarR family and demonstrate that zinc allosterically stabilizes DNA binding-competent ZitR in an atomic detail^52–55^. Of note, we concentrated on the dynamic of Zn^mono^–ZitR system and proved its role as a conformational transition state, which connects the DNA binding-incompetent state with the DNA binding-competent state. A previous study has proved that metal ions limit the location of AdcR DNA binding helices using NMR method^47^. Similarly, our preselection model emphasizes that zinc inhibits the motion of the α4 and α4′ helices and restrains their poses for DNA binding, in line with the results of NMR experiments. Crystal structure evidence and dynamic studies also provide clues for the structural stability and population shift effects brought by zinc^7,9,28^. Actually, it has been proved that allosteric regulation processes of prototypical MarRs are dynamic-driven and they are confined in the DNA binding-incompetent conformation^28,47^. In contrast, allosteric regulation in the metalloregulatory MarRs such as AdcR and ZitR yields the DNA binding-competent conformation, which may be explained by the different biological functions between prototypical and metalloregulatory MarRs^47^. The distinction of these discoveries may provide insights into the evolution of allostery, allosteric drug development and DNA recognition in transcription factor families^56,57^.

## Supplementary information

Supplementary information.
