# Supplementary material for: Zinc-mediated conformational preselection mechanism in the allosteric control of DNA binding to the zinc transcriptional regulator (ZitR)
Source: Sci Rep. 2020 Aug 6;10:13276. doi: 10.1038/s41598-020-70381-8 (PMC7413533; doi:10.1038/s41598-020-70381-8)
Supplement: Supplementary file 1 — Supplementary information. [file 41598_2020_70381_MOESM1_ESM.docx]

**Supporting Information**

**Zinc-Mediated Conformational Preselection Mechanism in the Allosteric Control of DNA Binding to the Zinc Transcriptional Regulator (ZitR)**

Xinheng He^1,2^, Duan Ni^1^, Hao Zhang^1^, Xinyi Li^1^, Jian Zhang^1,2,3^, Qiang Fu^4*^, Yaqin Liu^3*^, Shaoyong Lu^1,2,3*^

^1^Research Center for Marine Drugs, State Key Laboratory of Oncogenes and Related Genes, Department of Pharmacy, Renji Hospital, Shanghai Jiao Tong University, School of Medicine, Shanghai 200127, China

^2^Department of Pathophysiology, Key Laboratory of Cell Differentiation and Apoptosis of Chinese Ministry of Education, Shanghai Jiao Tong University, School of Medicine, Shanghai, 200025, China

^3^Medicinal Chemistry and Bioinformatics Center, Shanghai Jiao Tong University, School of Medicine, Shanghai, 200025, China

^4^Department of Orthopedics, Shanghai General Hospital, Shanghai Jiao Tong University, School of Medicine, Shanghai 200080, China

*Contact: Medicinal Chemistry and Bioinformatics Center, Shanghai Jiao Tong University, School of Medicine, Shanghai, 200025, China

E-mail addresses: [lushaoyong@yeah.net](mailto:lushaoyong@yeah.net) (S. Lu), [liuyaqin7811@126.com](mailto:liuyaqin7811@126.com) (Y. Liu)

Department of Orthopedics, Shanghai General Hospital, Shanghai Jiao Tong

University, School of Medicine, Shanghai 200080, China

E-mail address: [johson.f@163.com](mailto:johson.f@163.com) (Q. Fu)


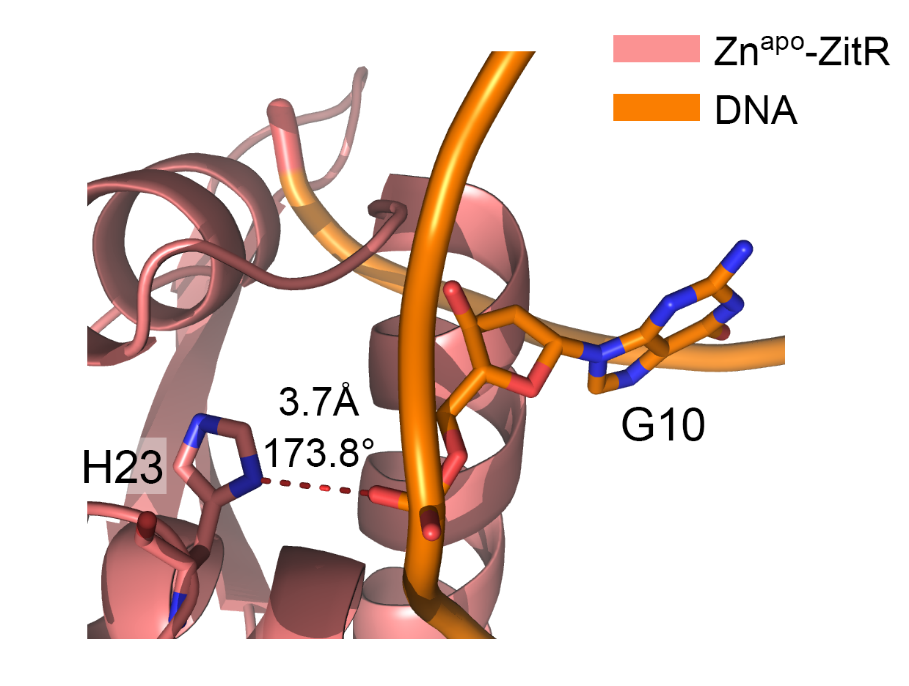


**Figure S1.** The hydrogen bond between G10 and H23 in Zn^apo^-ZitR−DNA is shown as a red dashed line.


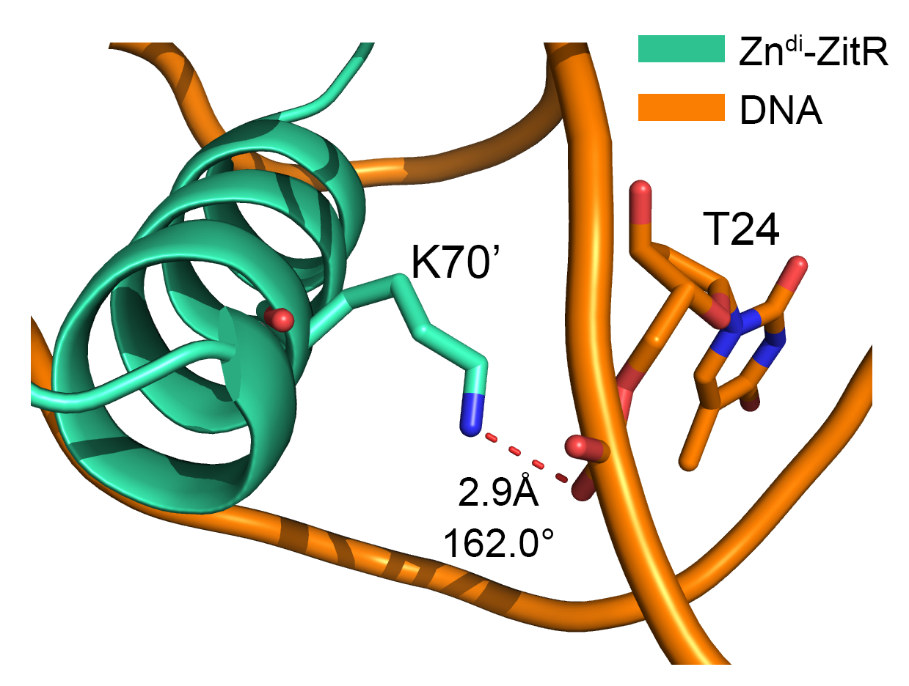


**Figure S2.** The hydrogen bond between T24 and K70’ in Zn^di^-ZitR−DNA is shown as a red dashed line.


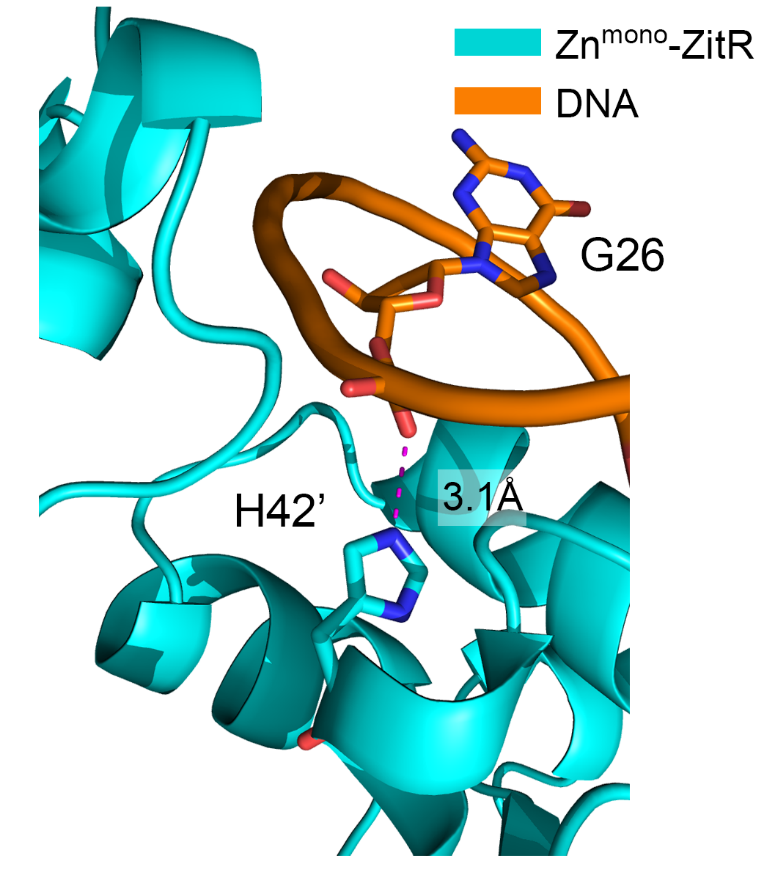


**Figure S3.** The ionic interaction between G26 and H42’ in Zn^mono^-ZitR−DNA is shown as a magentas dashed line.


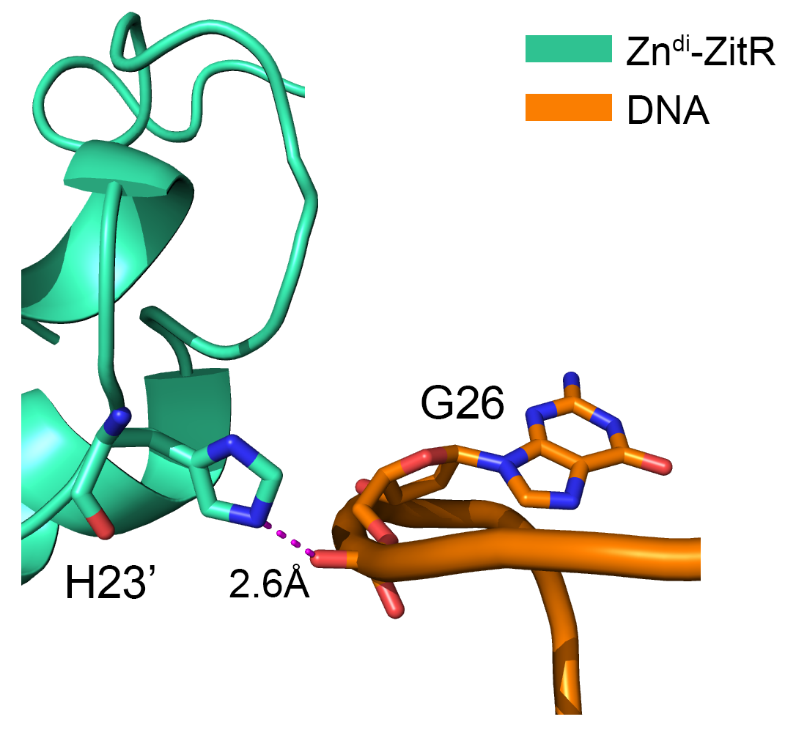


**Figure S4.** The ionic interaction between G26 and H23’ in Zn^di^-ZitR−DNA is shown as a magenta dashed line.


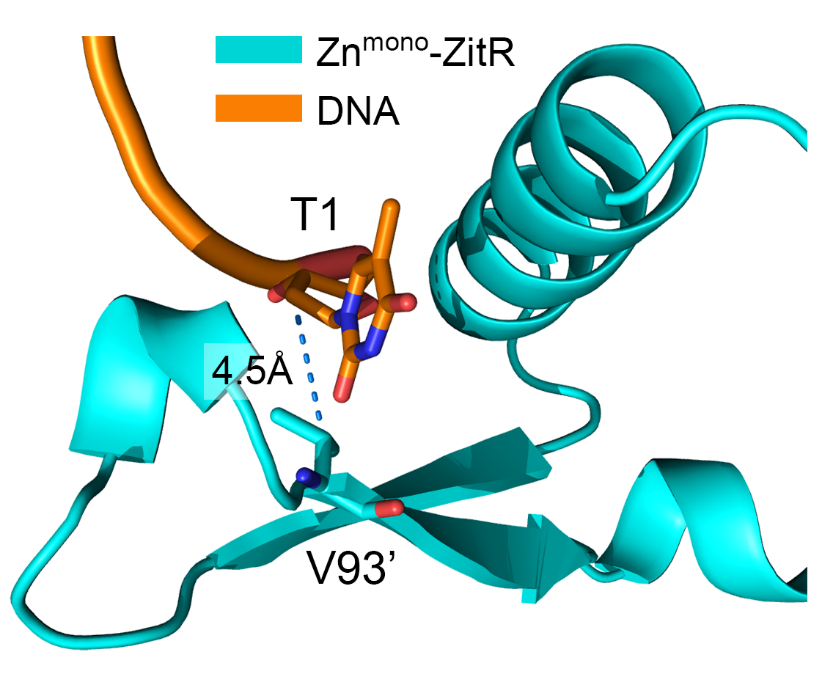


**Figure S5.** The hydrophobic contact between T1 and V93’ in Zn^mono^-ZitR−DNA is shown as a blue dashed line.


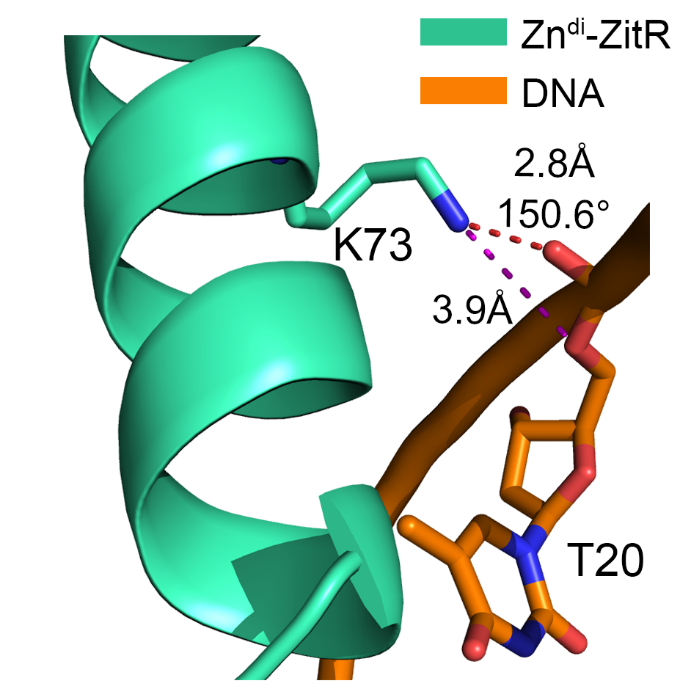


**Figure S6.** The ionic interaction (magenta) and hydrogen bond (red) between T20 and K73 in Zn^di^-ZitR−DNA.


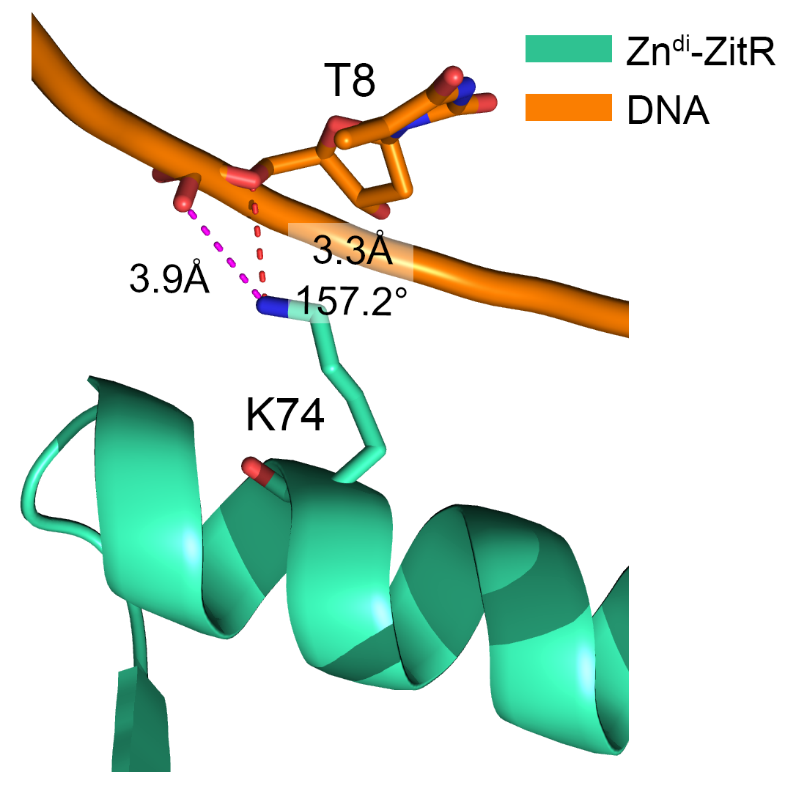


**Figure S7.** The ionic interaction (magenta) and hydrogen bond (red) between T8 and K74 in Zn^di^-ZitR−DNA.


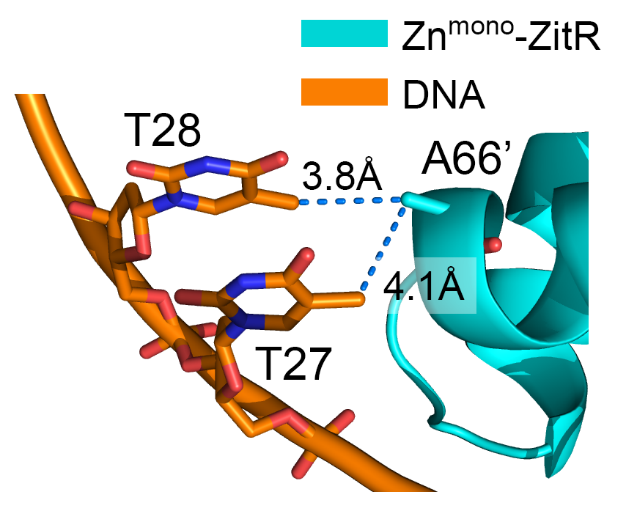


**Figure S8.** The hydrophobic contacts among T27, T28, and K74 in Zn^mono^-ZitR−DNA.


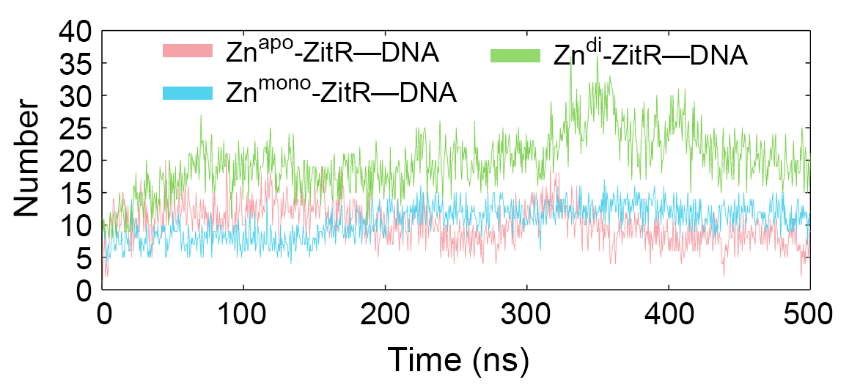


**Figure S9.** The number of hydrogen bonds between ZitR and DNA in three systems over time.


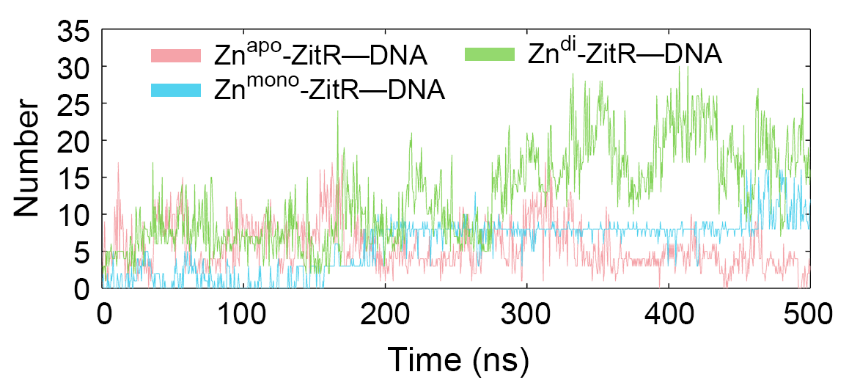


**Figure S10.** The number of ionic interactions between ZitR and DNA in three systems over time.


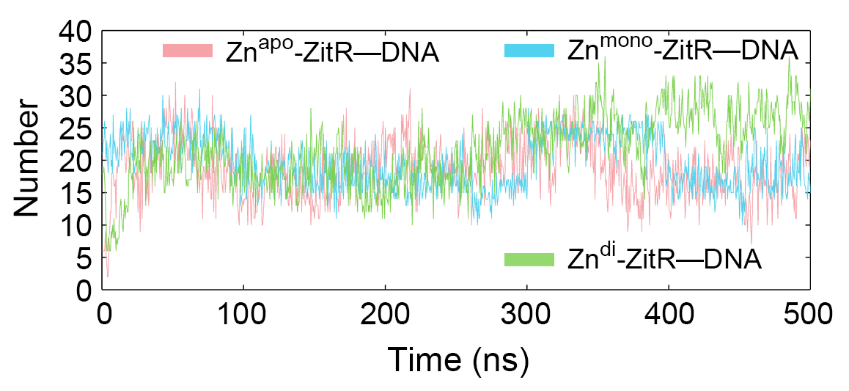


**Figure S11.** The number of hydrophobic contacts between ZitR and DNA in three systems over time.
